# Plasma Lipids as Biomarkers for Alzheimer's Disease: A Systematic Review

**DOI:** 10.7759/cureus.12008

**Published:** 2020-12-10

**Authors:** Mehak Agarwal, Safeera Khan

**Affiliations:** 1 Internal Medicine, California Institute of Behavioral Neurosciences & Psychology, Fairfield, USA

**Keywords:** alzheimers disease, cognitive impairment, plasma lipid biomarkers, cholesterol, brain cholesterol metabolism

## Abstract

Alzheimer's disease (AD) is caused by several risk factors leading to dementia. It’s diagnosis usually depends on clinical presentation and certain biomarkers in the cerebrospinal fluid (CSF). The brain has a high content of cholesterol and the metabolism of cholesterol in the brain can be associated with beta-amyloid plaques formation, which is seen in Alzheimer’s disease. Given these implications, we studied if plasma lipid levels can vary in Alzheimer's disease and if these can be used as biomarkers to diagnose and predict the progression of Alzheimer's disease. Certain mutations in the brain cholesterol transport receptors and proteins and their association with Alzheimer's were also studied. This systematic review abides by the Preferred Reporting Items for Systematic Reviews and Meta-Analyses (PRISMA) guidelines. We searched multiple databases, such as Pubmed, Google Scholar, Pubmed central, ScienceDirect, Web of Science, and Medline with the help of keywords like Alzheimer's disease, cognitive impairment, plasma lipid biomarkers, cholesterol, brain cholesterol metabolism separately and in combination with each other. We collected 49 quality appraised articles on the association between plasma lipids and Alzheimer's disease and the genetic mutations in alleles related to cholesterol metabolism and Alzheimer's disease by applying the inclusion and exclusion criteria. Based on the finding of the studies reviewed, we found an association between plasma lipids, polymorphisms in genes associated with cholesterol transport, and Alzheimer's disease. Increased serum low-density lipoprotein (LDL-C), triglycerides (TG), total cholesterol (TC), sphingolipids, 24S hydroxycholesterol (24S-HC), 27O hydroxycholesterol (27O-HC) was associated with Alzheimer's. Decreased high-density lipoprotein (HDL-C) and phospholipids were noticed. Genetic mutations in apolipoprotein E (ApoE), apolipoprotein B (ApoB), apolipoprotein A (ApoA), ATP binding cassette transporter 1 (ABCA1), ATP binding cassette transporter 7 (ABCA7), amyloid precursor protein (APP), cytochrome P450 family 46 subfamilies A member 1 (CYP46A1), presenilin 1 (PSEN1), presenilin 2 (PSEN2) are also associated with increased risk of Alzheimer's disease. This study found an association between plasma lipids and Alzheimer's, proving that plasma lipids can be used as biomarkers for early diagnosis of Alzheimer's disease. It may also help predict the prognosis and stage the disease severity. Further studies are needed to find out the exact mechanism behind these changes.

## Introduction and background

Alzheimer's disease and other causes of dementia constitute an increasing challenge in the health care system worldwide. There are approximately 50 million people currently living with dementia [1]. Alzheimer's disease is associated with a high mortality rate; it is the seventh leading cause of death in older people [2]. Alzheimer's disease (AD) was discovered based on the findings of an autopsy, which suggested atrophy of the brain cortex [3].

Alzheimer's disease is characterized by memory loss and cognitive impairment due to degeneration in the brain. It's the most common type of dementia. It's a progressive disease beginning with mild cognitive impairment resulting in memory loss, language, and thinking ability. Mild cognitive impairment is the phase between the cognitive impairment normally seen in the elderly and the cognitive decline due to complex conditions causing dementia. Pathologically, Alzheimer's disease is due to the building up of beta amyloid-forming plaques in the brain cortex and deposition of phosphorylated tau protein in the neurofibrillary tangles [3]. 

Both genetic and non-genetic risk factors can cause Alzheimer's disease. Ageing is the most important risk factor. Other causes include cerebrovascular disease, increased blood pressure, increase insulin resistance in type two diabetes mellitus, bodyweight, metabolic syndrome, smoking, traumatic brain injury, plasma lipid levels, diet, intellectual activity, and decreased physical activity [4]. Research focusing on the genetic component of Alzheimer's disease shows that Individuals with the apolipoprotein E4 (ApoE4) allele are at a higher chance of the disease [5-7]. Early-onset Alzheimer's disease is usually caused due to genetic variants in genes coding for amyloid precursor protein (APP) or presenilin (PSEN 1 and PSEN 2) [8-10]. Late-onset Alzheimer's is not known to be due to mutations in these genes. 

Currently, the diagnosis of Alzheimer's disease is made based on the clinical presentation of the patients and the laboratory diagnosis of three biomarkers in the cerebrospinal fluid (CSF): amyloid-beta 42, total tau, and phospho-tau, out of which beta-amyloid 42 is the most sensitive biomarker [11]. Biomarkers of cognitive decline help to detect the biochemical and pathological changes of AD in the cerebrospinal fluid. Cerebrospinal fluid is present in the brain, and hence a sample of the cerebrospinal fluid would help us evaluate the onset and progression of the disease. But a collection of a cerebrospinal fluid sample is an inconvenient process requiring a lumbar puncture, which is not routinely done in primary care, geriatric care, or psychiatry. This has led to the discovery of other biomarker molecules found in the plasma, which can help us evaluate Alzheimer's disease without the need for invasive, expensive tests and can also be done as large-scale screening tests. These biomarkers are plasma lipids: high-density lipoprotein (HDL-C), low-density lipoprotein (LDL-C), Total serum cholesterol, total cholesterol/HDL ratio, triglycerides, 24S-hydroxycholesterol (24S-HC), lipoprotein A, phospholipids, and sphingolipids. It is better to test this entire panel of biomarkers, as a single biomarker will not help detect a complex condition, such as Alzheimer's disease. Many recent research pieces have found a link between plasma lipids and Alzheimer's disease [12-15]. The brain has very high lipid content; hence changes in the brain phospholipids and cholesterol levels can easily lead to pathology in the brain [16,17]. 

The blood-brain barrier (BBB) prevents the entry of circulating cholesterol into the brain. The brain produces cholesterol from the astrocytes, which is later converted to 24S-hydroxycholesterol. 24S-Hydroxycholesterol can cross the blood-brain barrier and is excreted via the bile from the plasma [5]. In the case of hyperlipidemia, increased plasma cholesterol leads to the formation of free radicals, which disrupt the blood-brain barrier and lead to increased cholesterol in the brain. Cholesterol plays a key role in amyloidogenesis in the brain, causing more beta-amyloid plaques formation and leading to neurodegeneration. ApoE4 is a carrier for cholesterol transport; hence an individual carrying an allele of ApoE4 is prone to developing Alzheimer's [5,6]. Many other genetic mutations of the receptors and transporters involved in cholesterol metabolism and transport, such as ATP binding cassette transporter 1 and 7 (ABCA1, ABCA7), APP have caused Alzheimer's disease [8].

This study aims to show the relationship between plasma lipids and Alzheimer's disease, to demonstrate if increased plasma cholesterol increases the risk for Alzheimer's disease. It will help us understand if these plasma lipid biomarkers can be used as (1) diagnostic biomarker - to evaluate the risk of the onset of AD and (2) monitoring biomarkers - to estimate the progression of the condition. We will also discuss the various genetic variants associated with the onset of Alzheimer's disease.

## Review

Method

This systematic review strictly follows the Preferred Reporting Items for Systematic Reviews and Meta-Analyses (PRISMA) guidelines [18]. For data collection, we searched multiple electronic databases, such as PubMed, Google Scholar, Medline, ScienceDirect, PubMed Central, and Web of Science and the website of Neuropathology. Data were collected in September 2020. We used keywords such as "Alzheimer's disease," "cognitive impairment," "plasma lipid biomarkers," "cholesterol," "brain cholesterol metabolism" separately and in combination with each other. We found 3,417 articles with the help of these keywords. The articles' screening was done by going through the topics and abstracts and keeping the ones relevant to our research question. Inclusion and exclusion criteria were applied, and articles were further narrowed down to relevant ones. We did the quality appraisal of all the reference articles by following guidelines, and good quality forty-nine articles were kept. 

Inclusion Criteria

Study selection included the following criteria: studies conducted in English, on humans over 40 years of age, in the last 20 years, that were relevant to our topic and research question, peer-reviewed, full texts, including these study types - clinical trials, observation studies (case-control, cohort, and cross-sectional studies), systematic review, meta-analysis and literature review.

Exclusion Criteria

Grey literature, books, letter to editor, editorials, duplicate and overlapping studies, in vitro or animal studies. 

Result

**Figure 1:**
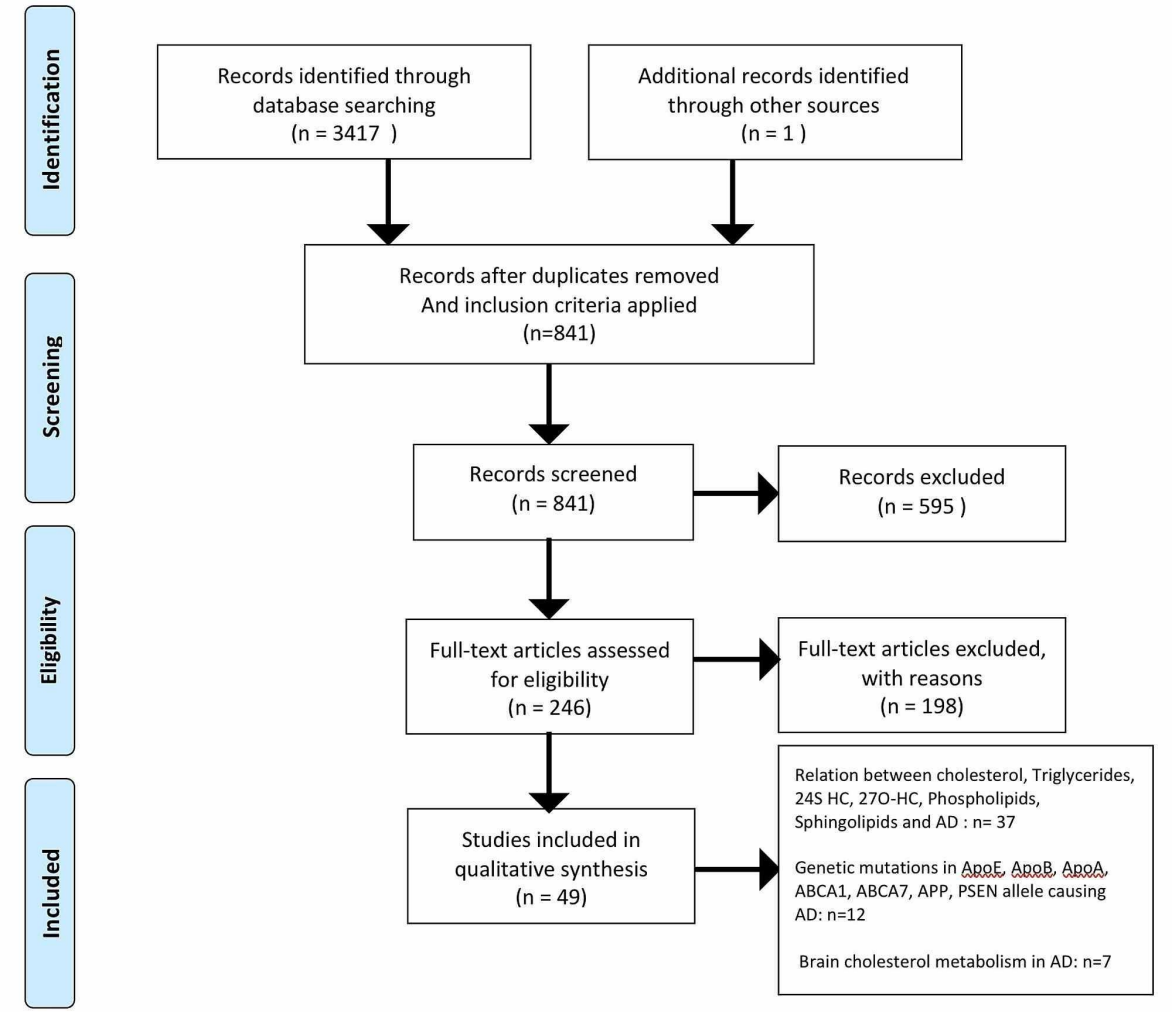
PRISMA flow chart PRISMA: Preferred Reporting Items for Systematic Reviews and Meta-Analyses; AD: Alzheimer's disease, APO: apolipoprotein, APP: amyloid precursor protein; 24S HC: 24S hydroxycholesterol; 27O-HC: 27O hydroxycholesterol; PSEN: presenilin; ABCA1 and ABCA7: ATP binding cassette transporter 1 and 7

A total number of 3,417 studies were identified from the databases. Filters applied based on inclusion criteria (full-text studies in English, last 20 years, on humans, clinical trials, all types of reviews, observational studies), and studies were filtered and reduced to 246. Screening of the articles was done, and relevant studies kept - 54. Quality appraisal was done for all studies, and the number of studies included reduced to 48. These included 11 systematic reviews/meta-analyses, 12 literature reviews, two randomized control trials, 13 case-control studies, seven cohort studies, and three cross-sectional studies. One article from the website of Neuropathology was included. A total of 49 articles were studied [1-49]. This study includes 37 studies that proved the relationship between increased plasma cholesterol, triglycerides, 24S hydroxycholesterol, 27O hydroxycholesterol, sphingolipids and phospholipids, and Alzheimer’s disease. Twelve studies prove that genetic mutations in ApoE, ApoB, ApoA, ABCA1, ABCA7, APP, and PSEN 1 and PSEN2 alleles are associated with AD. Seven studies explain the metabolism of cholesterol in the brain and the pathology associated with AD (Figure 1).

Discussion

We studied 49 previously published articles, some stressing the association between Alzheimer’s disease and plasma lipids and some about the genetic variants of various cholesterol transporters causing AD. In this study, we found that a dysregulation of brain lipid homeostasis can lead to cognitive disorders such as Alzheimer's disease. The exact pathway by which certain mutations happen is still not clear and more research is needed to confirm that. 

Cholesterol Metabolism in the Brain and Pathology Related to AD

The brain has a rich lipid content. The majority of cholesterol in the central nervous system (CNS) is present in two places; one is the oligodendrocytes of the myelin sheath, and the other is the plasma membrane of astrocytes and neurons. Myelin comprises 70% lipids containing cholesterol, sphingolipids, and phospholipids [19]. Cholesterol, sphingolipids are essential components of the neuronal plasma membrane present in the form of lipid rafts. It participates in signal transduction, neurotransmitter release, synaptogenesis, and membrane trafficking [11,20]. It is believed that the developing brain produces a high level of cholesterol, but this gradually reduced in the adult brain. The adult brain synthesizes cholesterol by the astrocytes, then transported to the neurons to carry out its functions. 

Cholesterol metabolism in the brain depends on brain cells' synthesis, transport across the cells, BBB, and catabolism [5]. Neurons depend on glial cells (astrocytes) for cholesterol. Astrocytes synthesize cholesterol from acetyl CoA by the HMG-CoA reductase enzyme. The transport of cholesterol to neural cells takes place with the help of ApoE which is also synthesized by astrocytes. The ApoE-cholesterol complex is transported with ABCA-1 and is taken up by the neurons via endocytosis through the LDL receptor-related protein (LRP-1) [5]. The endocytosed cholesterol in the neuron is further hydrolyzed to form free cholesterol. Free cholesterol undergoes esterification with acyl-coenzyme A cholesterol acyltransferase to form cholesterol esters stored in the neuron's cytoplasm. Some of the free cholesterol also controls the expression of cholesterol synthesizing enzymes and lipoprotein receptors such as liver X receptors (LXRs). These LXRs lead to increased expression of the ABCA1, thus mediating the transport of cholesterol from cells to apolipoproteins [5,6]. There is no degradation mechanism for any excess cholesterol in the brain. The excess cholesterol is converted to 24S-HC by the cytochrome P450 family 46 subfamilies A member 1 (CYP46A1) enzyme and is transported out of the brain to the blood-brain barrier [21]. 

The pathognomonic feature of AD is the building up of beta-amyloid plaques in the brain. In a healthy brain, the beta-secretase/beta-site amyloid precursor protein cleavage enzyme -1 (BACE-1) and gamma-secretase are present in the lipid rafts of the neuronal plasma membrane and cause cleavage of APP forming beta-amyloid. However, in AD, increased cholesterol lipid rafts induce raft clustering and enhance BACE-1 and APP interaction leading to increased beta-amyloid production [5,20,22]. 

**Figure 2:**
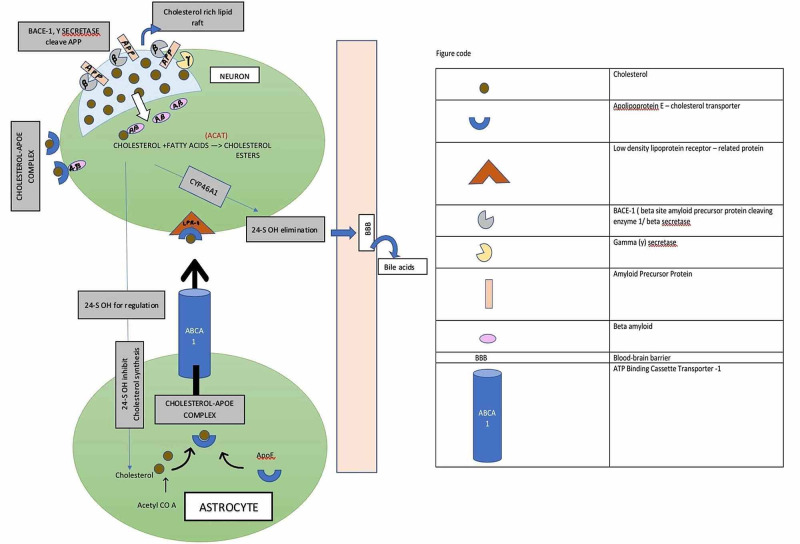
Demonstrating the process of synthesis, metabolism, and transport of brain cholesterol

The BBB maintains CNS homeostasis by regulating the transport of solutes between blood and brain. The BBB allows diffusion of oxygen and carbon dioxide freely, although lipophilic molecules such as cholesterol enter through receptors or channels. The brain lipid nature's homeostatic balance helps in controlled beta-amyloid production by APP cleavage, maintains the receptor channels, vesicle formation, secretion, signaling, inflammation, oxidation, membrane biosynthesis, and remodeling. Dysregulation in the brain lipid environment attributes to disturbed BBB, abnormal APP processing, abnormal cytosis, signaling, increased inflammation, oxidation. Long term, these can result in neuronal death, leading to AD (Figure 2) [22].

The Relation Between Plasma Lipids and AD

Many researchers have found a link between cholesterol homeostasis and AD; however, the exact pathogenesis remains unclear. We know that brain is rich in cholesterol content. The cholesterol in the brain and periphery are two separate units as they are well separated by a blood barrier, which restricts the entry of peripheral cholesterol. The cholesterol present in the lipid rafts of plasma neuronal membrane causes cleavage of APP. In the case of increased cholesterol in the lipid rafts, cholesterol enhances the activity of BACE-1 and Gamma-secretase and causes cleavage of APP leading to increased beta-amyloid production. These beta-amyloid plaques are characteristic hallmarks of AD. Increased plasma LDL-C, TC, triglycerides (TG), and decreased HDL-C was associated with increased beta-amyloid plaques causing AD. A cohort study by Pappolla et al. emphasized the association between high plasma cholesterol and patients with AD [23]. They found that increased cholesterol in the plasma was leading to increased beta-amyloid production. Many other studies found the same association between cholesterol and beta-amyloid plaques in the brain [4,24]. Some studied proved that beta-amyloid isoforms 1-40, 1-42 are associated explicitly with plasma cholesterol [7,13]. The study by Iqbal et al. was a systematic review showing that increased LDL-C, TC, TG were increasing beta-amyloid production. In contrast, decreased HDL-C was associated with AD [24].

Apart from amyloid plaques, AD is also associated with atrophy of the left/right hippocampal and entorhinal cortex. These changes are seen in the early stage of AD even before symptoms could arise. A case-control study by Proitsi et al., including 300 patients, also found that increased plasma lipids led to amyloid plaques in the brain and hippocampal and entorhinal cortex atrophy patients with AD [25]. Another study by Wolf et al. also studied the hippocampal atrophy of the brain and found its link with cholesterol; however, it was only associated with HDL-C [26]. The role of HDL-C in the disease process of AD is controversial. HDL is found to reduce the build-up of beta-amyloid plaques, thus reducing inflammation. Many researchers studied this protective role of HDL-C. Formiga et al. included 321 patients in a cohort study and proved the association between decreased HDL and AD [27]. Few other studies also proved the same objective [26,28]. Physical activity can decrease HDL levels and has proven to improve symptoms and progression of AD by a randomized control trial of 170 patients [28]. 

Cholesterol and triglycerides are also known to be a predictive marker for cardiovascular diseases. These lipids build up and obstruct the arteries supplying the heart. A similar mechanism was assumed for AD. Scientists believed that AD happens due to the brain's poor oxygenation due to the lipids' clogged vessels. Some studies determined the relation between these serum lipids and AD and if it is similar to the cardiac risk profile. A cohort study of nearly 4000 subjects by Helzner et al. shows that plasma lipids are associated with AD and other vascular diseases [29]. However, the mini-mental state exam (MMSE) was not affected, and hence it is still unclear how the cardiac risk profile affects cognition [15,30]. The relation between cholesterol and low MMSE was seen in a study by Hall et al. [31]. 

Unlike the studies already discussed, some studies say that only TC has an association with AD. The theory remains unclear, though. A study by He et al. proved that LDL, HDL, and triglycerides levels remain normal and only increased TC was seen in AD [32]. He included 130 patients in his case-control study. Similar studies by other authors also proved the same concept [33,34]. These studies by Solomon et al. and Anstey et al. suggest that total cholesterol has a bidirectional association with AD. It was seen to rise to midlife, suggesting cognitive impairment, and later declined with age in patients with AD. Solomon et al. proved this by conducting a large-scale case-control study, including 1321 patients with AD and 1203 controls [34]. Some other studies also found that only a single lipoprotein- low-density lipoprotein was elevated in AD, serum levels of rest were normal.

LDL-C is seen to cause vascular and neurotoxic effects in the brain [35,36]. Another study by Zhon, a systematic review including nearly 6500 patients, found the same association; however, LDL-C was high in patients with AD mostly around 60-70 years of age, gradually reduced with ageing [37]. Some studies found an association between AD and only LDL-C, TC. Many hypotheses are present. Some say LDL-C, TC is associated with increased tau concentration, according to some LDL-C, TC cause increased amyloid build-up, and some found that LDL and TC disrupt the cell cycle [38]. A study by Liu et al., including around 2333 AD patients and 3615 healthy controls, also suggested the association between LDL-C, TC, and AD [39]. Two studies also found that cholesterol remains normal, and only the serum triglyceride level is increased in the case of AD [22,40]. 

It is known that de novo synthesis of cholesterol occurs in the brain, and any disruption in this mechanism can lead to AD. 24S Hydroxycholesterol is the elimination product of neuronal cholesterol that leaves the brain and enters the periphery by crossing the BBB. 24S-HC and 27O hydroxycholesterol in the plasma indicates the degree of beta-amyloid production, loss of active grey matter, phosphorylated tau accumulation, and brain atrophy, thus indicating AD [11]. A case-control study by Popp et al., including 200 patients, found an association between increased plasma 24S hydroxycholesterol, 27O hydroxycholesterol, and AD [41]. Another study also showed a similar association [30]. 

Another group of lipids associated with AD is the sphingolipids and phospholipids. Sphingolipids such as sphingomyelin, ceramide, sulfatide, and sphingosine are major constituents of the neuron's plasma membrane. They are present in the lipid rafts, hence have a role in enhancing the activity of BACE-1 and gamma-secretase that causes cleavage of APP, forming beta-amyloid [20]. Phospholipids such as phosphatidylcholine, plasmogens, phosphatidylinositol, too, are a part of the membrane-forming lipid [20]. An association between serum and CSF levels of sphingolipids and phospholipids and AD has been studied. A study by Wong et al. showed that CSF sphingomyelin levels increase in the prodromal stage in AD, CSF ceramide increases in AD, CSF sulfatide levels decrease in AD, CSF phospholipids levels also increase. Whereas in the blood, ceramide level increases, sphingomyelin levels decrease, and phospholipids decrease in AD [20]. Another study by Kosicek et al. also found similar results [17]. A case-control study by Costa et al. studied ten membrane phospholipids and their association with AD. These were found to decrease in AD (Table 1) [42]. 

**Table 1:** Summarizing the association between plasma lipids and AD LDL-C: low-density lipoprotein cholesterol; HDL-C: high-density lipoprotein cholesterol; TC: total cholesterol; TG: triglycerides; AD: Alzheimer's disease; APO: apolipoprotein; Aβ: beta-amyloid; MCI: mild cognitive impairment; BBB: blood-brain barrier; 24S HC: 24S hydroxycholesterol; 27O-HC: 27O hydroxycholesterol; PC: phosphatidylcholine; PLA2: phospholipase 2

	Author	Year of Publication	Type of Study	Purpose of Study	Intervention Studied	Result/Conclusion
1.	Saiz-Vazquez et al. [35]	2020	Meta-analysis	To determine the association of serum cholesterol and AD.	Total serum cholesterol, HDL-C, LDL-C, and serum triglycerides	The relation between increased LDL-C and AD was found. No association between TC, TG, HDL-C, and AD were found.
2.	Zhou et al. [37]	2020	A systematic review and meta-analysis	To determine the association of serum cholesterol and AD.	LDL-C was measured	Elevated LDL-C leads to AD. This association is more in patients 60–70 years of age and gradually declines with age.
3.	Iqbal et al. [24]	2020	Systematic review	To determine the association of serum lipids and AD	HDL-C, LDL-C, TG, TC	HDL-c was found to be low, whereas LDL-C, TG, TC were high in cases of AD.
4.	Bernath et al. [40]	2020	Cross-sectional study	To determine the association of triglycerides with AD	Serum TG	Serum TG was found to be high in cases of AD
5.	Jensen et al. [28]	2020	Randomized control trial	To determine if physical exercise influences the lipid profile and AD	HDL-C, TC, LDL-C, triglycerides	With an increase in physical activity, cholesterol was lower, thus decreasing the risk of AD.
6.	Chew et al. [22]	2020	Literature review	To determine the factors affecting lipid metabolism and the association between plasma lipids and AD.	Age, sex, race, diet, plasma lipids level	Decreased plasma HDL-C and increased triglyceride is associated with AD.
7.	Liu et al. [39]	2019	Meta-analysis	To determine the association between plasma cholesterol and AD	Total serum cholesterol, HDL-C, LDL-C, and serum triglycerides	Serum LDL-C and total cholesterol were found to be elevated in AD. No association between HDL-C and triglycerides was found.
8.	Wu et al. [38]	2019	Meta-analysis	To determine the association between plasma lipids and AD	HDL-C, TC, LDL-C	LDL-C, TC are increased in AD
9.	Costa et al. [42]	2019	Case-control study	To determine the association between phospholipids and AD, and the activity of PLA2 in the brain	Serum phospholipids, PLA2 by radio enzymatic assay	Ten serum phospholipids were assessed and found to decrease in AD; PLA2 activity was also decreased in the neuronal membrane in AD.
10.	Anstey et al. [34]	2017	A systematic review and meta-analysis	To determine the association between plasma lipids and AD in midlife	HDL-C, LDL-C, TC, TG	Increased TC in midlife is found to be associated with late-onset AD. No association between HDL and TG with AD was found.
11.	Proitsi et al. [25]	2017	Case-control study	To determine the association between plasma cholesterol and AD and the associated brain atrophy	Plasma lipids. Brain – left/right hippocampal area, entorhinal cortex	Increased plasma lipids were associated with AD, causing brain atrophy.
12.	Wong et al. [20]	2017	Literature review	To determine the progress in lipidomics research in AD with the help of mass spectrometry	Phospholipids, sphingolipids, and cholesterol	Increase total cholesterol increase the risk of AD. Sphingolipids: in CSF – sphingomyelin levels increase in prodromal AD, ceramide increases in AD, sulfatide decreases in AD. As blood – ceramide level increases, sphingomyelin decreases in AD. Phospholipids: increases in CSF and decreases in blood in AD.
13.	Wang et al. [11]	2016	Meta-analysis	To determine if 24S-HC and 27 O HC are biomarkers for AD	24S-HC, 27S-HC	24S-HC and 27O-HC are sensitive biomarkers for AD diagnosis
14.	He et al. [32]	2016	Case-control study	To determine the association of plasma lipids with MCI in the elderly	Total cholesterol, HDL-C, LDL-C, serum triglycerides were measured	TC was elevated in the elderly with AD. No association between LDL-C and AD was found. HDL-C and TG were negatively related to AD.
15.	Hall et al. [31]	2014	Systematic review	To determine if increased cholesterol in AD is gender-specific	Total serum cholesterol	Males were found to have higher total Cholesterol in AD
16.	Toro et al. [44]	2014	Cohort study	To determine the association of total cholesterol in AD and MCI and the ApoE genotype	Total cholesterol	Increased TC is seen in patients with MCI and AD even before symptom scan arise. This is independent of the APOE genotype.
17.	Lukiw [49]	2014	Literature review	To determine if cholesterol and 24 hydroxycholesterol trafficking in the brain causes AD and CYP46A1 gene effects on AD	Cholesterol and 24 hydroxycholesterol, beta-amyloid plaques, CYP46A1 genotyping	Beta-amyloid plaques, which is characteristic of AD, were found with increased CSF 24 hydroxycholesterol, a mutation in the CYP46A1 gene causes dysfunctional cholesterol metabolism
18.	Reitz and Mayeux [4]	2014	Literature review	To determine the genetic and non-genetic risk factor of AD	Plasma and CSF lipid markers, genetic mutations, age, physical activity, BMI, cerebrovascular disease.	Cholesterol was found to be increased in cases of AD.
19.	Popp et al. [41]	2013	Case-control study	To determine the association between cerebral and extracerebral cholesterol and its relation with AD.	Plasma and CSF cholesterol, cholesterol precursors, 24 hydroxycholesterols, and 27 hydroxycholesterols were measured.	Cholesterol synthesis was found to be de novo in the brain. Twenty-four hydroxycholesterols were found to increase in the case of AD.
20.	Kosicek and Hecimovic [17]	2013	Literature review	To determine the association between phospholipids and AD	Sulfatide, ceramide, sphingomyelin, PC	Sphingomyelin increases in the prodromal stage of AD, ceramide increases, Sulfatide decreases, and PC metabolites decreased in AD.
21.	Formiga et al. [27]	2012	Cohort study	To determine the association of HDL-C levels with mental, physical activity, and cognition	HDL-C	Decreased HDL-C was only found to improve functional aspect but not cognitive performance
22.	Helzner et al. [29]	2009	Cohort study	To determine the role of vascular risk factors in AD.	Total cholesterol, HDL-C, LDL-C	High total cholesterol and LDL-C levels and diabetes cause an increased incidence of AD, thus confirming that vascular risk factors play a role in AD.
23.	Mamo et al. [13]	2008	Case-control study	To investigate if lipoproteins are bound to Aβ isoforms	Fasting state lipoproteins	The majority of plasma triglycerides, VLDL, and IDL, was bound to Aβ_1–40_ isoform
24.	Solomon et al. [33]	2007	Case-control study	To determine the association between TC and AD	TC	Serum TC increases in midlife in AD and later decreases with age.
25.	Raygani et al. [ 46]	2006	Case-control study	To determine the association between plasma lipids, ApoE polymorphism, and with AD	HDL-c, LDL-C, TC, APOB, APOE4, APOA1	Apolipoprotein e4 is associated with AD. Also, decreased apoA1, HDL-C, increased Apo B, increased LDL-C, TC is seen in AD.
26.	Solfrizzi et al. [30]	2006	Literature review	To determine the association between biomarkers such as HDL-C, TC, LDL-C, homocysteine, lipoprotein A, inflammatory cytokines, and AD	HDL-C, LDL-C, TC, triglycerides, homocysteine, LP(A), cytokines	All these biomarkers were found to increase in association with AD
27.	Sabbagh et al. [15]	2005	Case-control study	To determine the association between plasma lipids and AD	HCL-C, LDL-C, TC, TG, TOTAL/HDL RATIO	Increased TC, TG, LDL-C was associated with AD. HDL-C and total/HDL ratio remains normal in AD.
28.	Wolf et al. [26]	2004	Randomized control trial	To determine the association between hippocampal volume (presumptive index of AD) and plasma lipids	Plasma HDL-C, LDL-C, TC were studied	HDL-C was found to be associated with hippocampal volume in the brain.
29.	Pappolla et al. [23]	2003	Cohort study	To determine the association between amyloid plaques and hypercholesterolemia	Amyloid deposits using imaging and immunohistochemistry and serum cholesterol	Association was seen between hypercholesterolemia and beta-amyloid plaques deposited in the human brain
30.	Kuo et al. [7]	1998	Case-control study	To determine if elevated LDL-C in AD is related to beta-amyloid 1-42	TC, HDL-C, LDL-C, lipoproteins, ApoB	A correlation was found between the levels of serum total cholesterol, LDL-C, and ApoB with only Aβ N-42 in AD, not Aβ N-40.
31.	Moroney et al. [36]	1999	Cohort study	To determine the effect of lipids on dementia with stroke	HDL-C, LDL-C, triglycerides, total cholesterol, lipoprotein A, APOE4.	Increased plasma LDL-C was found in patients with dementia with stroke, but its relationship with the ApoE4 allele was not established.

Genetic Mutations in Cholesterol-Related Genes and AD

Apolipoprotein E is produced by the astrocytes and is a carrier transport protein, shifting cholesterol from astrocytes to neurons. Apolipoprotein E also has an affinity for beta-amyloid in the presence of cholesterol. Some studies found that ApoE is required for the clearance of beta-amyloid. Thus, any mutation in this could lead to a decrease in the clearance and building up of plaques [6]. There are three isoforms of ApoE: ApoE2, ApoE3, ApoE4. ApoE3 is the commonest isoform present in the majority of the population; however, ApoE4 has the strongest established association with AD. Any individual homozygous for this allele and old can develop AD [6]. Since it has such a strong association, any mutations in ApoE4 can result in AD. According to studies, ApoE4 is an independent risk factor for AD [10]. A Cohort study by Kivipelto et al., including 1449 AD patients, suggests that ApoE 4 is an independent risk factor for AD [43]. Another cohort study by Toro et al. also found the same association [44]. Other isoforms of ApoE are not as closely related to causing AD as ApoE4. ApoE2 does not have any role in AD, and ApoE3 has protective effects against AD. A systematic review by Agarwal et al. suggests the above and found that all alleles of ApoE4 - ApoE 2/4,3/4,4/4 are associated with AD [45].

Some other types of apolipoproteins are also related to AD. ApoB is another lipoprotein associated with AD; the exact reason remains unclear. In the brain, most of the cholesterol is present in density similar to that of HDL-C and transported via ApoE or ApoA [5]. Any mutation in the ApoA gene can also cause AD. A study conducted by Raygani et al. found that apart from ApoE 4, increased ApoB and decreased ApoA1 are associated with Alzheimer's disease [46]. Early-onset AD has also been associated with mutations in the genes coded for enzymes and transporters involved in beta-amyloid metabolisms, such as APP, PSEN1, and PSEN2. APP cleaves to form beta-amyloid, and presenilin is a protein present in the gamma-secretase complex [8]. Studies have shown the association between AD and mutation in the gene of APP, PSEN1, PSEN2, and ApoB [47]. 

**Figure 3:**
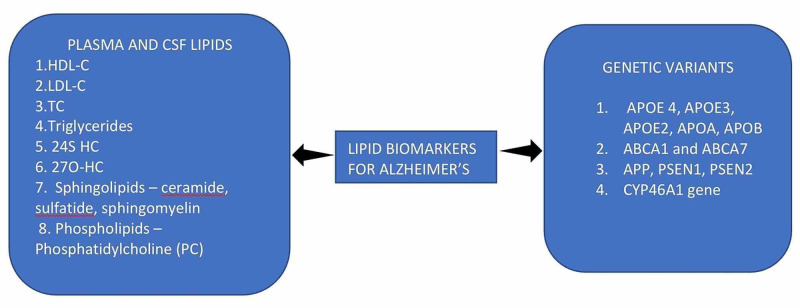
Plasma biomarkers of Alzheimer's disease and their genetic variants LDL-C: low-density lipoprotein cholesterol; HDL-C: high-density lipoprotein cholesterol; TC: total cholesterol; TG: triglycerides; Apo: apolipoprotein; APP: amyloid precursor protein; 24S HC: 24S hydroxycholesterol; 27O-HC: 27 O hydroxycholesterol; PSEN: presenilin; ABCA1 and ABCA7: ATP binding cassette transporter 1 and 7; CYP46A1: cytochrome P450 family 46 subfamilies A member 1

Some studies have found the association between another cholesterol transporter - ABCA1 and AD. ABCA1 is essential for the cholesterol efflux from the CSF to the serum. ABCA1 is also found to reduce beta-amyloid accumulation. Lack of gene/defect in the gene of ABCA1 is associated with building up of amyloid plaques, causing AD [16]. A study by Li et al. studied the association between ABCA1 and Cholesterol efflux causing AD [48]. The same study also found the association between another genotype, ABCA7, and AD. By the metabolism of cholesterol, we have studied that cholesterol does not cross BBB; hence it is metabolized to 24S hydroxycholesterol with the help of the enzyme CYP46A1 and further eliminated via the BBB. Any mutation in the gene coding for CYP46A1 can lead to defects in elimination, causing increased cholesterol and more amyloid plaques leading to AD. A study by Lukiw et al. found the association between 24S-HC, cholesterol synthesizing enzyme, and AD (Table 2, Figure 3) [49].

**Table 2:** Summarizing association between mutations in cholesterol metabolism protein and Alzheimer's disease LDL-C: low-density lipoprotein cholesterol; HDL-C: high-density lipoprotein cholesterol; TC: total cholesterol; TG: triglycerides; AD: Alzheimer's disease; Apo: apolipoprotein; Aβ: beta-amyloid; APP: amyloid precursor protein; MCI: mild cognitive impairment; BBB: blood-brain barrier; 24S HC: 24S hydroxycholesterol; 27O-HC: 27O hydroxycholesterol; PSEN: presenilin; ABCA1 and ABCA7: ATP binding cassette transporter 1 and 7; CYP46A1: cytochrome P450 family 46 subfamilies A member 1

Author	Year of Publication	Type of Study	Purpose of Study	Intervention Studied	Result/Conclusion
Wings et al. [8]	2019	Case-control study	To determine if plasma cholesterol and genetic variants of various cholesterol transport proteins are associated with AD.	Total cholesterol, LDL-C, HDL-C, TG, and genetic variants in ApoB, ApoE, APP, PSEN1 and PSEN2.	Primary outcome: the relationship between plasma cholesterol and AD was seen. Secondary outcome: AD was associated with mutations in APOE, APP, PSEN1, PSEN2, and APOB.
Jeong et al. [6]	2019	Literature review	To determine if APOE induced cholesterol dysfunction affects the various brain cells and causes AD.	APOE4, APOE3, APOE2 alleles	APOE ɛ4 one or more alleles increase the risk of AD
Li et al. [48]	2017	Case-control study	To determine if the ABCA7 genotype of cholesterol transport protein it's associated with sporadic AD	ABCA7 genotyping	ABCA7 genotype was associated with lipid homeostasis and AD.
Yassine et al. [16]	2016	Cross-sectional study	To determine if ABCA-1 mediated cholesterol efflux is affected in patients with AD and MCI	CSF's role in cholesterol transport was assessed using a BHK cell line that expressed the ABCA1 transporter.	In case of MCI and AD, the role of CSF was impaired. ABCA1‐mediated cholesterol efflux does not take place as normal, leading to AD.
Agarwal et al. [45]	2014	Meta-analysis	To determine the association between ApoE alleles and the risk for AD	APOE genotyping	All genotypes of the ApoE e4 allele, increase the risk of AD, although the ApoE e2, e3 alleles protect from AD.
Toro et al. [44]	2014	Cohort study	To determine the association of total cholesterol in AD and MCI and the ApoE genotype	Total cholesterol	High TC levels are associated with AD but are independent of the APOE genotype.
Lukiw [49]	2014	Literature review	To determine if cholesterol and 24 hydroxycholesterol trafficking in the brain causes AD and CYP46A1 gene effects on AD	Cholesterol and 24 hydroxycholesterol, beta-amyloid plaques, CYP46A1 genotyping	Beta-amyloid plaques, which is characteristic of AD, were found with increased CSF 24 hydroxycholesterol, a mutation in the CYP46A1 gene causes dysfunctional cholesterol metabolism
Caramelli et al. [47]	1999	Case-control study	To determine the relationship between plasma lipids and AD.	VLDL, HDL-C, LDL-C, triglycerides, ApoB, lipoprotein (a)	Significantly higher Apolipoprotein B levels were found in AD patients, whereas the concentration of lipoprotein (a) and plasma lipids was not statistically different. This shows that APOE may not be the only transporter associated with AD.
Raygani et al. [46]	2006	Case-control study	To determine the association between plasma lipids, ApoE polymorphism, and with AD	HDL-C, LDL-C, TC, APOB, APOE4, APOA1	Apolipoprotein e4 is associated with AD. Also, decreased ApoA1, HDL-C, increased ApoB, increased LDL-C, TC is seen in AD
Panza et al. [10]	2006	Literature review	To determine the association between TC, 24 S hydroxycholesterol, LDL-C, Lp(A), ApoE levels with AD	TC, 24S HC, LDL-C, Lp(A), APOE	TC, LDL-C, Lp(A) was found to be elevated in patients with AD. No consistent association between ApoE and AD have been found in this study
Martins et al. [5]	2006	Literature review	To understand cholesterol mechanism, the relation between APOE allele and AD, determine convergence risk factors for AD and CAD	APOE ɛ4 allele	APOE ɛ4 one or more alleles increase the risk of AD. A decrease in APO A1 is associated with AD.
Kivipelto et al. [43]	2002	Cohort study	To determine the association between APOE ɛ4 allele and AD	APOE genotype	APOE ɛ4 alleles are associated with AD

Limitations

Even though the study clearly shows the relation between AD and plasma cholesterol, there are many aspects of cholesterol metabolism in the brain that are still not clearly understood. Different studies included in this study have different theories about AD pathology due to high plasma cholesterol. The exact reasons for polymorphism in many genes and associated AD also remain unclear. We were also unable to find if these biomarkers are altered by gender and physical activity. Only studies published in English were included; we did not include articles that were in other languages. Case reports, case series, letter to the editor, editorials were not included. Similarly, studies that did not find any association between AD and cholesterol were also excluded.

## Conclusions

We studied many articles on the association between AD and plasma lipids. Based on those, we found that levels of plasma cholesterol, triglycerides, sphingolipids, phospholipids are altered in AD, and genetic variants of the various cholesterol metabolism-related proteins also lead to AD. The brain is made up of high lipid content, and thus any alteration in the cholesterol metabolism in the brain can cause dysregulation of the brain lipid homeostasis. Cholesterol is associated with the beta-amyloid build-up in the brain, thus increased plasma cholesterol results in increased beta-amyloid plaques, which is pathognomonic of AD. We also found out that ApoE4 and mutations in many other transporters of cholesterol in the brain are linked with increasing the chances of AD. This article thus points out all the lipid-associated risk factors causing AD. This will help us improve our knowledge and scope of the disease. Plasma lipids biomarkers can also be studied by a simple blood test making diagnosis and prediction of AD much easier. However, more advanced studies and research must understand the exact pathology behind this, as brain and peripheral cholesterol are two different entities and separated by a strict BBB.
